# Network analysis reveals causal relationships among individual background risk factors leading to influenza susceptibility

**DOI:** 10.1038/s41598-025-15131-4

**Published:** 2025-08-21

**Authors:** Akihide Terada, Kenji Fujimoto, Kazuyoshi Kise, Kenta Fujiwara, Eiichiro Uchino, Yutaka Mizuma, Yoshinori Nishioku, Kenzo Takahashi, Ken Itoh, Tatsuya Mikami, Koichi Murashita, Shigeyuki Nakaji, Yukihiro Fujita, Yasushi Okuno, Yoshinori Tamada

**Affiliations:** 1 Department of Endocrinology and Metabolism, Graduate School of Medicine, Hirosaki University, Aomori, Japan; 2 Department of Healthy Life Expectancy Extension Science, Graduate School of Medicine, Hirosaki University, Aomori, Japan; 3 Department of Precision Health Care, Graduate School of Medicine, Hirosaki University, Aomori, Japan; 4 Taisho Pharmaceutical Co., Ltd., Tokyo, Japan; 5 Department of Biomedical Data Intelligence, Graduate School of Medicine, Kyoto University, Kyoto, Japan; 6 Department of Stress Response Science, Biomedical Research Center, Graduate School of Medicine, Hirosaki University, Aomori, Japan; 7 Department of Preemptive Medicine, Innovation Center for Health Promotion, Graduate School of Medicine, Hirosaki University, Aomori, Japan; 8 Research Institute of Health Innovation, Hirosaki University, Aomori, Japan; 9 Department of Medical Data Intelligence, Research Center for Health-Medical Data Science, Graduate School of Medicine, Hirosaki University, Aomori, Japan

**Keywords:** Influenza, Bayesian network analysis, Causal inference, Health checkup data analysis, Personalized medicine, Biomedical engineering, Disease prevention, Public health

## Abstract

Influenza is a serious infectious disease that spreads rapidly and causes numerous deaths worldwide. Although the biological and environmental risk factors for influenza susceptibility have been extensively studied, individual risk factors, such as medical history and lifestyle, and their complicated relationships remain unclear. In this study, we aimed to reveal individual-specific causal relationships among influenza risk factors using large-scale health checkup data collected from residents of a rural community. To this end, we quantitatively represent the relationships between each factor for individuals using a Bayesian network. Our network revealed multiple causal pathways from each health checkup item to influenza onset/non-onset. Furthermore, we performed a cluster analysis based on the individual network profiles to reveal the characteristics of the participants susceptible to influenza. We identified five distinct types of participants: hyperglycemia, pneumonia, hectic and sleep-deprived, malnutrition, and allergies. Our results highlight the importance of personalized influenza prevention strategies based on individual backgrounds. To the best of our knowledge, this is the first study to identify individual-specific risk factors and comprehensive causal relationships for disease onset. Thus, our network-based analysis may contribute to the development of personalized disease prevention and treatment approaches.

**Supplementary Information:**

The online version contains supplementary material available at 10.1038/s41598-025-15131-4.

## Introduction

Influenza is an infectious respiratory disease caused by influenza viruses A and B. It usually results in mild and nonspecific symptoms, such as fever, sore throat, rhinorrhea, cough, headache, muscle aches, and fatigue, but it can sometimes lead to fatal pneumonia. It is estimated that 1 billion people are infected annually worldwide, with related deaths ranging from 291,000 to 646,000^1^. Despite widespread knowledge regarding the prevention and treatment of influenza, this disease continues to pose a significant threat to human health.

Influenza has been extensively studied to identify susceptible risk factors from both environmental and biological perspectives. From an environmental perspective, influenza viruses can survive longer and become more infectious in environments with lower temperatures and humidity^2–5^. Furthermore, high population densities in urban areas and frequent contact between people in enclosed spaces such as schools and public transportation facilitate the spread of influenza^6^. Air pollution, which impairs the respiratory defense system, is another factor increasing the risk of influenza. Indoor ventilation is critical because poor air circulation facilitates the spread of the viruses^7^. In addition, even in the same environment, some individuals may be more susceptible to influenza than others. In other words, personal biological background factors may affect influenza infections. Various studies have revealed such factors, including the diversity of immune responses^8^, occupation^9,10^, type I allergies^11^, and infection prevention measures^12^. However, to the best of our knowledge, no study has comprehensively analyzed the relationships among these factors.

In this study, we analyzed large-scale health checkup data to reveal multiple backgrounds related to influenza susceptibility and the relationships among them. Specifically, we used data collected from the Iwaki Health Promotion Project (IHPP)^13^ conducted by Hirosaki University, Japan. The IHPP annually performs a health checkup that targets approximately 1,000 community-dwelling residents aged 20 years and older and measures more than 3,000 parameters.

These parameters include well-known risk factors for influenza onset, such as younger age, occupation, and medical history^8^.

First, as a basic analysis, we compared the parameters between influenza-affected and non-affected participants and conducted a logistic regression analysis to identify factors associated with influenza onset.

Furthermore, to reveal the causal relationships among backgrounds and differences between individuals, we applied a Bayesian network (BN) analysis to the IHPP data. A BN is a probabilistic graphical model suitable for representing the causal relationships between multiple variables, and is particularly effective for exploratory causal discovery in high-dimensional observational data. We estimated the structure of the BN using IHPP data to extract the *causal pathways* that lead to influenza onset. In addition, by performing cluster analysis on the profiles of the estimated network, we identified five distinct groups of influenza-affected individuals: hyperglycemia, pneumonia, hectic and sleep-deprived, malnutrition, and allergy. This shows our BN analysis effectively identified individual-specific factors leading to influenza onset and may contribute to personalized disease prevention and treatment.

## Results

### Subject background & data preparation

To identify multiple backgrounds associated with susceptibility to influenza and the relationships between them, we used data collected during the IHPP health checkup conducted in 2019 (hereinafter, 2019 IHPP data). We examined data from 1,062 of the 1,065 participants (Table 1), covering 2,090 checkup items. Influenza onset/non-onset was determined using a questionnaire that asked whether the participants had experienced influenza onset in the past year (see details in Materials and Methods).

**Table 1:** Background of the participants.

	All	Non-onset	Onset
Number of analyzed participants	1,062	941	121
Gender (male / female)	435 / 627 (41% / 59%)	392 / 549 (42% / 58%)	43 / 78 (35% / 65%)
Age (Mean $\:\pm\:$ SD)	52.67 $\:\pm\:$ 15.25	53.05 $\:\pm\:$ 15.15	49.85 $\:\pm\:$ 15.73
Height (Mean $\:\pm\:$ SD)	161.72 $\:\pm\:$ 8.90	161.76 $\:\pm\:$ 8.85	161.46 $\:\pm\:$ 9.37
Body weight (Mean $\:\pm\:$ SD)	60.63 $\:\pm\:$ 12.53	60.78 $\:\pm\:$ 12.59	59.61 $\:\pm\:$ 12.17
BMI (Mean $\:\pm\:$ SD)	23.06 $\:\pm\:$ 3.64	23.10 $\:\pm\:$ 3.65	22.77 $\:\pm\:$ 3.60

We first selected the items possibly related to influenza onset using two selection methods: one based on machine learning and the other based on the experts’ opinions provided by the co-authors who were involved in pharmaceutical research or specialized in health science. In the machine learning-based approach, we constructed a machine learning model and selected 124 items that significantly contributed to the prediction (see more details in Materials and Methods). Additionally, 203 items were selected based on expert opinion. Among these, 66 items overlapped, resulting in a total of 261 unique items. In addition, we applied a data-cleaning procedure, including the removal of items with many missing values or high correlations with other items (see details in Materials and Methods). A total of 96 items were removed in this process. Finally, 165 items were used for analysis (Supplementary Fig. S1).

### Basic analysis

As a basic analysis, we identified the items that were significantly different between influenza onset and non-onset groups. Details on the definition of influenza onset are provided in Supplementary Materials (Section S1: Data Preparation). We conducted a bootstrap method with 1,000 iterations to compute the 95% confidence intervals of the differences between the two groups for each item. This analysis was performed to understand the magnitude and direction of each factor’s association with influenza onset/non-onset. In addition, to reduce the effects of multiple hypothesis comparison, we corrected the significance level using Dunn-Šidák method^14^. As a result, we identified variables whose 95% confidence intervals did not cross 0 (Table 2).

**Table 2:** Items significantly different between influenza onset and non-onset groups.

Domain	Item	Non-onset	Onset	Averaged difference [95% CI]	Susceptibility to influenza*
Background	Age	53.04 ± 15.16	49.85 ± 15.73	-3.19 [-6.07 to -0.30]	Young
	History of pneumonia	0.05 ± 0.2	0.13 ± 0.33	0.08 [0.02 to 0.14]	Having history and/or complications
	Use of over-the-counter drugs	0.10 ± 0.30	0.19 ± 0.39	0.09 [0.02 to 0.16]	Using
	Degree of skin itchiness^†^	11.69 ± 16.76	17.31 ± 19.36	5.63 [2.35 to 8.90]	High
Environment	Number of people living together	3.82 ± 1.65	4.23 ± 1.91	0.41 [0.09 to 0.73]	Many
	Sleeping hours^†^	6.95 ± 1.14	6.64 ± 1.07	-0.30 [-0.52 to -0.09]	Short
	Weekly hours for personal errands	5.93 ± 4.67	6.89 ± 4.32	0.96 [0.08 to 1.84]	Long
	Weekly hours for commuting	2.97 ± 6.32	4.62 ± 9.41	1.65 [0.36 to 2.94]	Long
	Weekly hours for watching TV	19.18 ± 16.27	15.84 ± 10.75	-3.34 [-6.36 to -0.31]	Short
Blood test	Dihomo-γ-lylolenic acid	1.23 ± 0.28	1.29 ± 0.32	0.06 [0.01 to 0.11]	High
	Glycoalbumin	14.55 ± 1.70	14.99 ± 3.36	0.45 [0.07 to 0.82]	High
	Docosahexaenoic acid	126.23 ± 46.78	115.16 ± 48.13	-11.07 [-19.97 to -2.17]	Low
	Pentosidine	26.21 ± 11.62	29.71 ± 35.03	3.50 [0.46 to 6.55]	High
	Na	141.45 ± 1.78	140.84 ± 2.02	-0.61 [-0.95 to -0.26]	Low
	Cl	104.61 ± 1.95	104.06 ± 2.18	-0.55 [-0.92 to -0.17]	Low
	C3	102.23 ± 17.45	99.03 ± 18.40	-3.20 [-6.53 to 0.13]	Low
	C4	23.97 ± 6.18	22.31 ± 5.40	-1.66 [-2.82 to -0.51]	Low
	EPA	67.16 ± 45.79	56.19 ± 40.95	-10.97 [-19.55 to -2.39]	Low
	EPA/AA ratio	0.33 ± 0.25	0.27 ± 0.24	-0.05 [-0.10 to -0.01]	Low
Nutrient intake	Intake of persimmon	5.41 ± 1.77	5.84 ± 1.58	0.43 [0.10 to 0.76]	Low
	Intake of fresh lettuce and cabbage	25.49 ± 21.78	21.16 ± 15.35	-4.33 [-8.34 to -0.32]	Low
	Intake of seasonings and spices	267.94 ± 144.55	239.23 ± 137.86	-28.71 [-55.96 to -1.45]	Low
	Intake of Vitamin B12	9.77 ± 6.36	8.37 ± 4.78	-1.40 [-2.58 to -0.23]	Low
	Intake of n-6 fatty acid (C22:5)	8.65 ± 6.43	7.29 ± 4.90	-1.36 [-2.55 to -0.17]	Low
Others	FEV1.0%	80.65 ± 6.85	82.15 ± 7.15	1.50 [0.18 to 2.81]	High
	Cerebrovascular disease risk rate	83.14 ± 116.20	61.32 ± 91.52	-21.82 [-43.45 to -0.19]	Low
	Ankle-brachial pressure index	9.41 ± 89.83	-12.01 ± 96.18	-21.42 [-38.66 to -4.19]	Low

* Susceptibility to influenza means how particular item contributes to increase the risk of influenza onset.

Variables marked with † are statistically significant after Dunn–Šidák correction for multiple hypothesis comparison^14^.

We then performed logistic regression analysis with influenza onset/non-onset as the objective variable and 165 items remaining after data preparation as explanatory variables. The estimated values of the regression coefficients and their 95% confidence intervals were calculated. To identify influenza-related items, we identified the items whose confidence intervals of the odds ratio did not cross 1 (Table 3).

**Table 3:** Influenza-related items identified through logistic regression analysis.

Domain	Item	Odds ratio [95% CI]	Susceptibility to influenza
Background	History of pneumonia	2.88 [1.55 to 5.35]	Having history and/or complications
	Use of over-the-counter drug	2.07 [1.25 to 3.41]	Using
Environment	Number of people living together	1.15 [1.03 to 1.28]	Many
	Sleeping hours	0.78 [0.66 to 0.93]	Short
Blood test	Dihomo-γ-lylolenic acid	2.08 [1.08 to 3.99]	High
	Glycoalbumin	1.10 [1.01 to 1.19]	High
	Na	0.83 [0.74 to 0.92]	Low
	Cl	0.87 [0.79 to 0.96]	Low
	C4	0.95 [0.92 to 0.99]	Low
	EPA/AA ratio	0.35 [0.13 to 0.90]	Low
Nutrient intake	Intake of persimmon*	1.17 [1.04 to 1.33]	Low
	Intake of vitamin B12	0.96 [0.92 to 0.99]	Low
	Intake of n-6 fatty acid (C22:5)	0.96 [0.92 to 0.99]	Low

*Note that the item “Intake of persimmon” is higher for the lower amount of intake.

### Network analysis

Basic analysis has identified various factors associated with influenza onset. However, the complex relationships between these factors have not yet been revealed. Each health checkup item can be influenced by a complex relationships that ultimately lead to outcomes such as influenza onset. A network is a graphical representation of such relationships among variables, where each variable is represented as a node, and connections such as causal or associative relationships are shown as edges (Supplementary Figure S2). It can help us understand the *causal pathways*, that is, the sequences of factors that lead to influenza onset/non-onset. To reveal such network, we conducted a BN analysis.

**Fig. 1:**
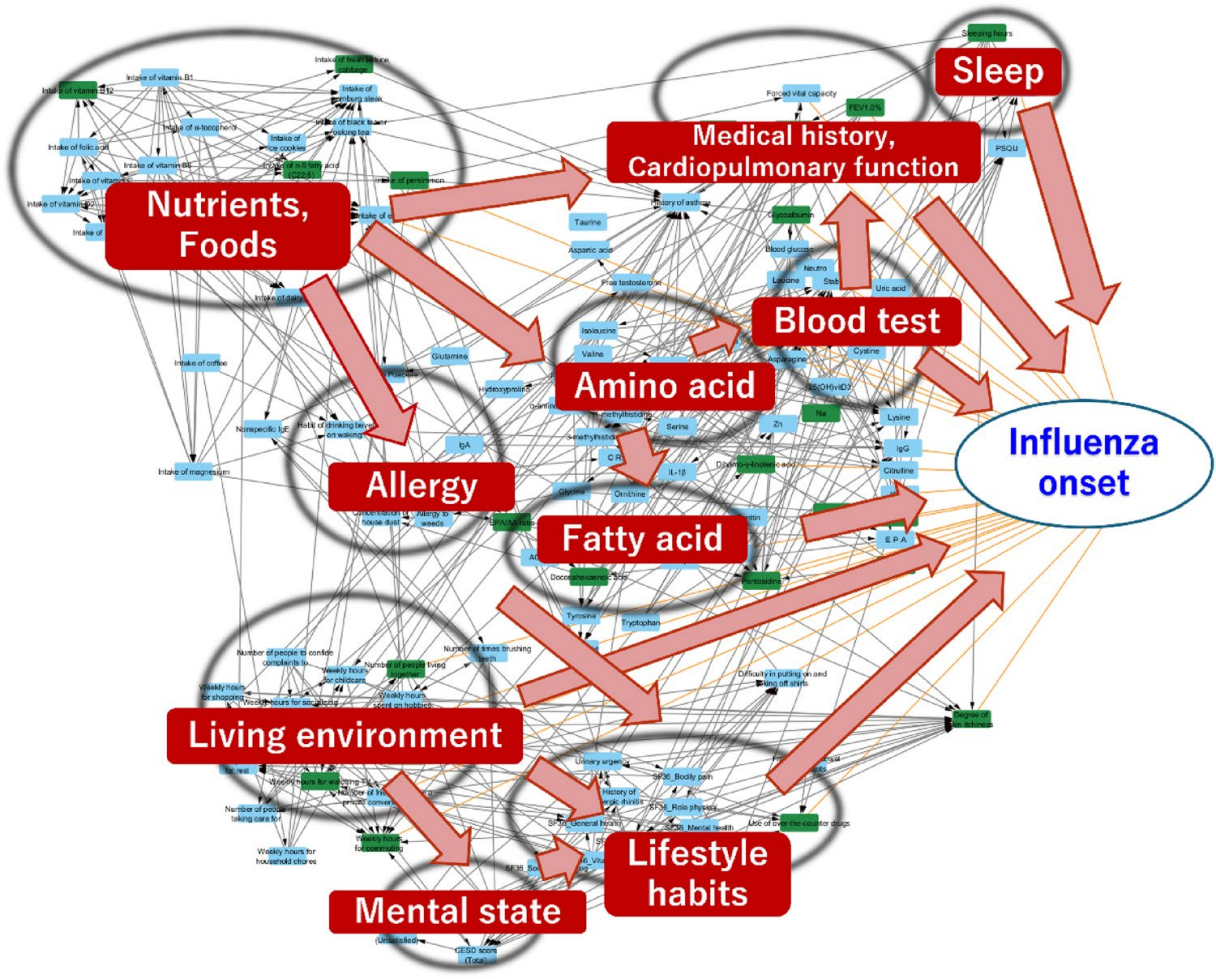
A network leading to influenza onset obtained through our network structure estimation. A brief interpretation of groups of items is overlayed on the network. The raw network diagram without overlayed boxes is available in Supplementary Fig. S3. Items represented as green nodes were also identified in our basic analysis. Orange edges are direct connections toward “Influenza onset.”

A BN is a network model that visualizes complex cause-and-effect relationships among the variables in a network. Using the 2019 IHPP data, we estimated the structure of the BN using a $\:B$-spline nonparametric regression algorithm (described in Materials and Methods). We then pruned the low-importance pathways based on the PathRC value of each pathway, which is the average relative contribution value of each edge included in the pathway (see Materials and Methods for details). Consequently, we obtained the network shown in (Fig. 1). Figure 1 shows that “Medical history, Cardiopulmonary function” and “Sleep” directly lead to influenza onset. “Nutrients and Foods” lead to influenza onset via “Blood test” and “Medical history, Cardiopulmonary function.” We can also find the pathways from “Nutrients and Foods” to influenza onset via “Allergy” and “Lifestyle habits.” The factors identified in the basic analysis, such as living environment, history of pneumonia, and sleep duration, were directly linked to influenza onset. Additionally, the causal pathways from these factors to influenza onset, as discussed in the previous section, can be found in the network. Supplementary Table S1 shows the main pathways from the factors identified in the basic analysis to influenza onset.

### Clustering of all participants

Figure 1 shows that multiple causal pathways lead to influenza onset. Generally, the pathways leading to influenza onset vary among individuals. However, a BN is an average structure statistically estimated using an entire dataset, and it is not possible to interpret which part of the structure is important for each individual.

To overcome this limitation, we attempted to determine how different the personalized networks were. We used the relative contribution (RC) values, that is, the quantified contribution of each parent node (variable) to a specific child node (variable), to profile each participant’s network (see Materials and Methods for details). Hierarchical clustering of all participants was performed based on RC values.

**Fig. 2:**
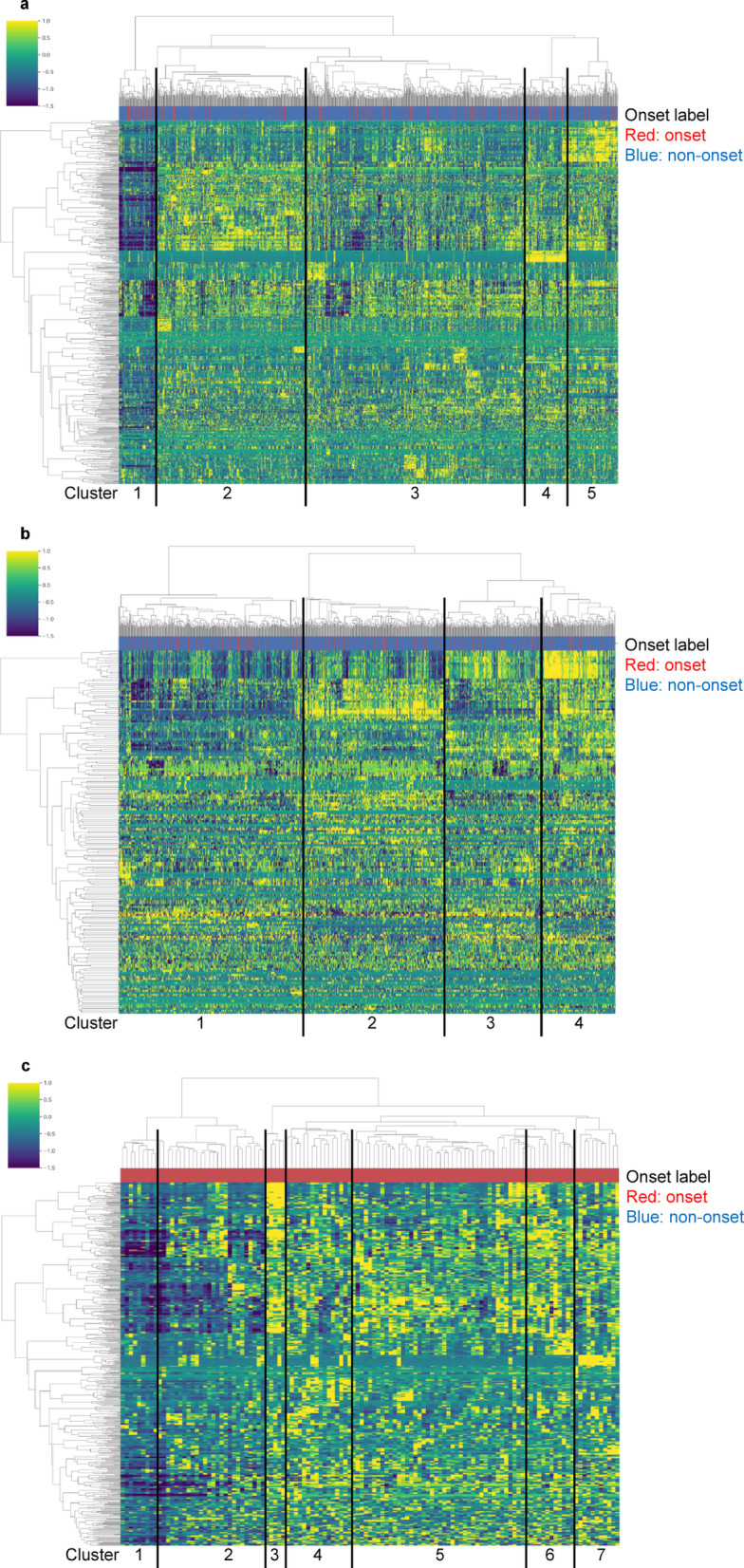
Heatmaps showing the results of hierarchical clustering. Each column corresponds to a participant. (**a**) The results of clustering of all participants based on RC values. The participants could be divided into five major clusters. (**b**) The results of clustering without RC values. Although the participants were classified into several clusters, there were no significant differences in onset rates or features. (**c**) The results of clustering of participants with influenza based on RC values. The participants could be divided into 7 major clusters.

Figure 2a shows the clustering results. The participants were classified into five major clusters. As shown in Table 4, the influenza onset rate for each cluster was higher in Cluster 1 (first left in Fig. 2a), with 28.4% (23/81 participants), than in the other clusters; it was lower in Cluster 2 (second from the left side of Fig. 2a), with 7.2% (23/319 participants). Comparing the onset rate of Cluster 1 with other clusters, the odds ratio was 3.6. Comparing the onset rates of Clusters 1 and 2, the odds ratio was 5.1. Therefore, our RC value-based clustering method successfully extracted populations with high and low onset rates.

**Table 4:** Influenza onset rate in each cluster identified in hierarchical clustering based on RC values.

Cluster	Non-onset	Onset	Total
1	58 (71.6%)	23 (28.4%)	81
2	296 (92.8%)	23 (7.2%)	319
3	411 (88.6%)	53 (11.4%)	464
4	79 (87.8%)	11 (12.2%)	90
5	96 (89.7%)	11 (10.3%)	107
Independent	1 (100.0%)	0 (0.0%)	1
Total	941	121	1062

To extract the characteristics of Cluster 1, which had a high influenza onset rate, we performed an analysis similar to the basic analysis. We performed bootstrap method on each item between Cluster 1 and the other clusters and identified items whose confidence intervals did not cross zero (Supplementary Table S2). This analysis showed that 50/81 (61.7%) of the participants in Cluster 1 had a history and/or complications of pneumonia compared to only 9/980 (0.9%) outside of Cluster 1. In addition, sleep efficiency was low, and Pittsburgh Sleep Quality Index Japan^15^ scores, which reflect impaired sleep quality with higher values, were high. The glycoalbumin levels were also elevated in this cluster. Therefore, we considered Cluster 1 as a population with a history or complication of pneumonia, impaired sleep, and/or high glycemia-related indices. These factors were also identified in the basic analysis, suggesting they are also risk factors for influenza.

In addition, we applied logistic regression to the population other than Cluster 1, with influenza onset as the objective variable and the other variables as explanatory variables (Supplementary Table S3). A higher number of people living together, regular use of over-the-counter medications (which may represent the existence of allergies and/or diseases), lower levels of C4, Na, and Cl, and insufficient intake of persimmon were also identified in our basic analysis, suggesting they are also risk factors for influenza onset.

### Clustering of all cases using original data

Hierarchical clustering using RC values derived from the network analysis successfully extracted the characteristics of groups susceptible to influenza. To validate the effectiveness of the RC values, we performed hierarchical clustering on the original data before network analysis and confirmed whether the features could be extracted without using the RC values (Fig. 2b).

Although we classified the data into several clusters, the influenza onset rate in each cluster was uniform, and distinct features could not be extracted (Supplementary Table S4). The highest and lowest onset rates were 12.9% and 9.6%, respectively. The odds ratio between the clusters with the highest and lowest onset rates was 1.39 (95% confidence interval: 0.76 to 2.557), and since the confidence interval includes 1, the difference was not statistically significant. These results indicate that, without using RC values, it was difficult to extract distinct or relevant features associated with influenza onset. In contrast, our results indicate that hierarchical clustering using RC values was an effective approach for feature extraction.

### Clustering of participants with influenza

**Fig. 3:**
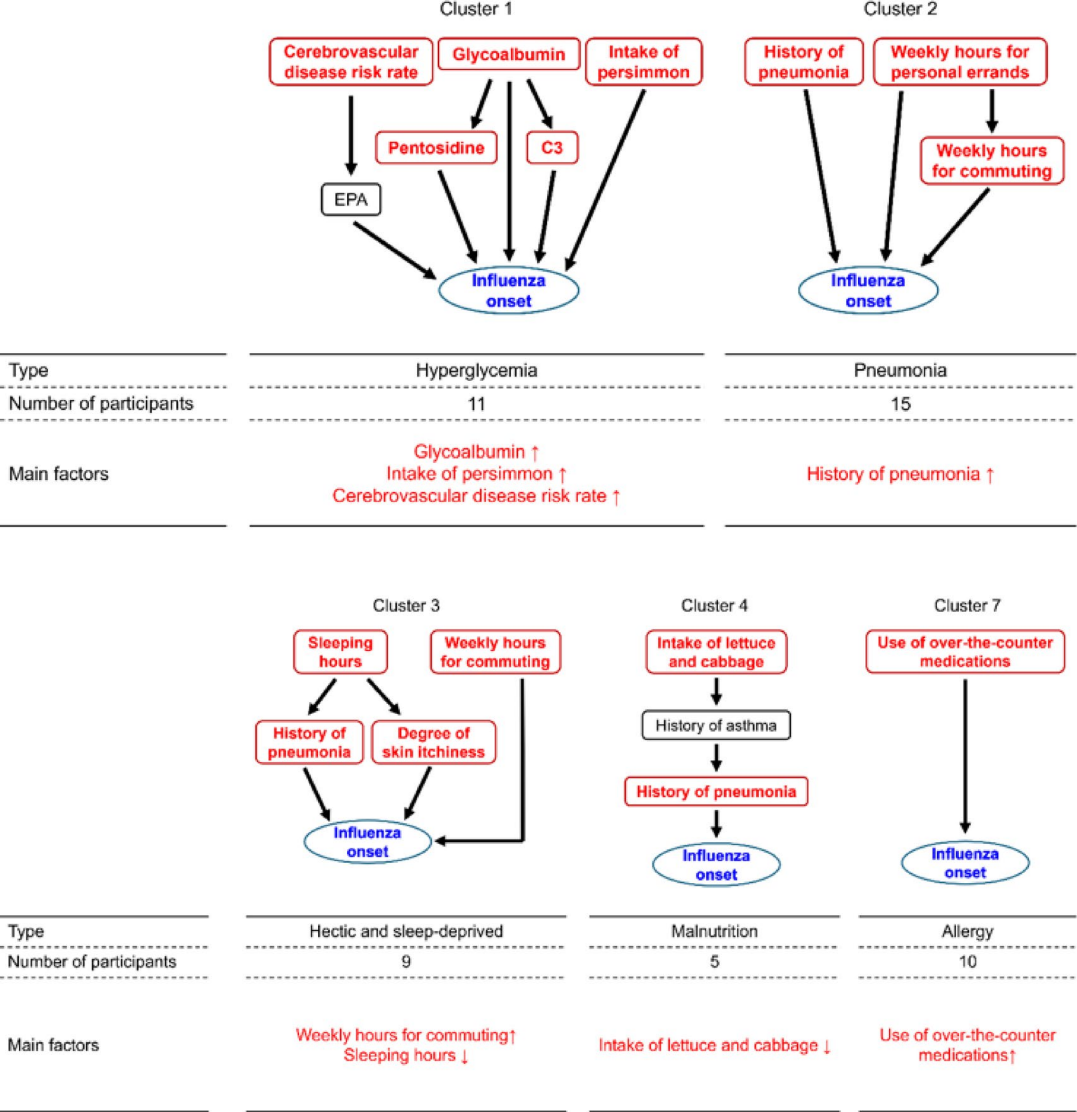
Main risk factors and pathways leading to influenza onset of each cluster. The odds ratio confidence intervals of red-colored items did not cross 1. In the row of main factors, $\:\uparrow\:$ and $\:\downarrow\:$ indicate that the higher and lower values of the factor contribute to the influenza onset, respectively. The interpretation of network structures can be difficult in some cases; the association between “lettuce” and “asthma” observed in Cluster 4 serves as one such example. Note that “use of over-the-counter medications” in Cluster 7 is considered to be the result of existing allergies and/or diseases.

The above analyses focused on determining the differences between influenza-onset and non-onset participants. Next, we investigated the differences within the group of influenza-onset participants. We performed hierarchical clustering of only influenza-onset participants based on RC values and identified 7 major clusters (Fig. 2c). We analyzed the distribution of each item by statistically comparing the values observed in each cluster against those in the other clusters (e.g., Cluster 1 vs. all others, Cluster 2 vs. all others, …) and obtained cluster-specific factors shown in Supplementary Table S5. Using these results, we found cluster-specific subgraphs and main factors specific to each cluster, as shown in Fig. 3 (see details in Materials and Methods).

Clusters 1–3 were clusters with major factors related to influenza onset, that is, poor plasma glucose-related indices, history and/or complications of pneumonia, and poor sleep-related indices, respectively. Participants with a history and/or complications of pneumonia were concentrated in Clusters 1 and 2, and no participants with a history or complications of pneumonia were found in the other clusters. Cluster 4 had a low intake of lettuce and cabbage. Cluster 5 was characterized by a degree of skin itchiness. Although skin itchiness is generally considered to be associated with allergic reactions, no other clear indicators suggestive of allergy were identified in this cluster. Cluster 6 was characterized by a shorter duration of watching TV, which was difficult to interpret in relation to influenza onset. Cluster 7 had higher allergy test values (weeds and cedars) than the other clusters. The cluster was characterized by a higher percentage of participants with allergic rhinitis.

In summary, our analysis classifies the influenza-onset population into hyperglycemia (Cluster 1), pneumonia (Cluster 2), hectic and sleep-deprived (Cluster 3), malnutrition (Cluster 4), and allergy (Cluster 7) types. As described above, Clusters 5 and 6 did not exhibit clear characteristics; therefore, we did not assign specific names to them. Furthermore, it could contribute to the development of personalized disease prevention and treatment approaches.

## Discussion

In this study, we analyzed health checkup data to identify factors associated with influenza onset and effective preventive measures for each individual. First, we discuss the validity of each factor shown in Tables 2 and 3 as a basic analysis. Our results suggest that participants with a history of pneumonia were more susceptible or less resistant to infection. Therefore, it was shown to be more relevant to influenza onset. Environmental factors such as the number of people living together and commuting time are also associated with the risk of exposure to the virus. A short sleep duration is known to increase the frequency of colds^16^. In our results, it was also associated with susceptibility to influenza. Complements 3 (C3) and 4 (C4) play important roles in the immune system and are therefore considered associated with susceptibility to influenza. They are proteins involved in the complement cascade, which enhances the ability of antibodies and immune cells to clear pathogens^17^. Hyperglycemia compromises the immune system against viruses by reducing dendritic cell function and impairing cytokine signaling^18,19^. High plasma glucose-related items, such as glycoalbumin and pentosidine, may reflect susceptibility to infection due to hyperglycemia. A lower EPA/AA ratio may reflect chronic inflammation, which affects susceptibility to infection^20^. In terms of allergy-related items, such as skin itchiness and regular use of over-the-counter medications (which may represent existing allergies and/or diseases), we consider that increased inflammation due to allergies and mouth breathing due to allergic rhinitis might increase susceptibility. A previous study^21^ showed that tannins in persimmons might act as antiviral agents. Therefore, persimmon intake may improve resistance to viruses and decrease susceptibility. Vitamin B12 is involved in the metabolism of lipids and proteins^22,23^ and may indirectly affect the immune system and susceptibility. While several of the identified factors were consistent with previous reports, not all factors could be clearly explained based on existing literature. These may reflect previously unreported associations. In the above discussion, most identified factors and contribution directions were consistent with previous reports. Therefore, our factor extraction method is considered appropriate.

In addition to the basic analysis, we identified the pathways leading to influenza onset through the accumulation of causal relationships between factors in categories such as “medical history, cardiopulmonary function,” “sleep,” “nutrients and foods,” “blood tests,” “allergies,” and “lifestyle.” Using the RC values derived from the network, we performed hierarchical clustering and identified a specific group that was more susceptible to influenza and its characteristics. Clusters characterized by poor sleep efficiency, glycemic indices, and a history and/or complications of pneumonia had significantly higher influenza onset rates than the other clusters. In addition, by clustering influenza-onset participants only, we successfully classified them into five major types: hyperglycemia, pneumonia, hectic and sleep-deprived, malnutrition, and allergies. Our findings highlight the importance of personalized health interventions to prevent influenza onset based on individual risk profiles, particularly in populations with pre-existing conditions such as pneumonia, sleep deprivation, and metabolic disorders.

This study has several limitations. First, genetic factors were not included among the analyzed variables, as genetic data were not available for this study. Such factors may play an critical role in individual susceptibility to influenza. Incorporating genetic factors in our BN analysis will be an important step toward personalized influenza prevention strategies. Second, we did not include any questions regarding influenza vaccination, as such items were not part of the original questionnaire. Including vaccination data could have provided insights not only into the effectiveness of the vaccine itself, but also into the characteristics of individuals who choose to get vaccinated. Such information is expected to be valuable for public health planning and interventions. Finally, while this study identified potential differences between individuals more or less susceptible to influenza, further research is needed to determine whether these findings can be applied prospectively or whether targeted interventions could help reduce susceptibility.

## Conclusion

The primary objective of this study was to identify the factors that make individuals susceptible to influenza. To this end, we demonstrated the effectiveness of a combination of statistical and network-based analyses for extracting and visualizing complex relationships between diseases and various health factors. While existing studies have mainly analyzed the relationship between a small number of factors and diseases, this is the first study to reveal the complex causal relationships between more than 100 items and their differences among participants. Therefore, this study may contribute to improved and personalized disease prevention strategies.

## Materials and methods

### Participants

We used the data collected during the IHPP health checkup in 2019. The health checkup and our study using the collected data were approved by the Ethics Committee of Hirosaki University Graduate School of Medicine (approval number 2020-046-4) and conducted in accordance with the Declaration of Helsinki. A total of 1,065 individuals living in the Iwaki district of Hirosaki City, Aomori Prefecture, Japan, participated in health checkups. All the participants provided written informed consent. We excluded the data of participants who did not answer the question about their history of influenza onset in the past year (*n* = 3). Finally, we analyzed the data of 1,062 participants.

### Influenza onset/non-onset status

Influenza onset/non-onset status in the past one year (2018–2019) was determined based on participants’ self-reports. The observed onset rate in this study was 121 out of 1,062 participants (11.4%), which is consistent with the general seasonal influenza incidence rate of approximately 10% in Japan.

### Health checkup items

From the 2019 IHPP data, we used 2,090 items divided into 7 domains.

- Dietary survey (brief-type self-administered diet history questionnaire^24^.
- Health questionnaire (such as medical history and lifestyle habits).
- Blood and urine tests.
- Body composition measurement.
- Four-limb blood pressure measurements.
- Pulmonary function tests.
- Locomotive syndrome and bone density tests.

### Item selection

To facilitate the interpretation of the results and reduce the computational costs of the BN analysis, we selected the essential background factors of influenza onset to narrow down the items. The item selection procedure is illustrated in Supplementary Fig. S1.

First, we adopted a machine-learning-based approach to select items related to influenza onset. Various machine learning models including support vector machines^25^, random forests^26^, light gradient boosting machines (LightGBM)^27^, and extreme gradient boostings (XGBoost)^28^ were trained to classify influenza onset/non-onset in parallel. The hyperparameters of each model were tuned using grid search. Then, the best-performing model was selected based on cross-validation. Next, we computed the permutation importance^29^, a popular criterion for estimating the importance of each variable in a prediction. We selected the items whose median permutation importance was greater than or equal to 0.0001, a threshold empirically determined based on computational limitations related to BN analysis and the manageable number of variables. We repeated these procedures 10 times with different random seeds and determined items related to influenza onset as the union of the 10 derived item sets. In the 10 iterations, the best-performing models were primarily LightGBM and XGBoost. As a result, 124 items were selected. We carried out the entire process using the DataRobot software^30,31^.

We then added 137 items previously known to be related to upper respiratory tract infections based on experts’ opinions. In addition, items containing many missing values (more than 20%) or those that were highly correlated with other items (Pearson’s correlation coefficient more than 0.5 or less than $\:-$0.5) were removed. Finally, we analyzed 165 items.

To show that the selected 165 items were related to influenza onset/non-onset, we trained a machine learning model to predict influenza onset/non-onset. We used LightGBM. The area under the ROC (receiver operating characteristic) curve of the model using the selected items was approximately 73%, which was 23% higher than the model using all items. Therefore, we considered the selected items were reasonable background factors for influenza onset.

### Basic analysis

We constructed logistic regression models for the basic analysis. The objective variable was influenza onset/non-onset, and the explanatory variables were the health checkup items selected in the previous section. In addition, we performed bootstrap method on each item to determine whether there was a significant difference between the influenza onset and non-onset groups. We used the Python, Statsmodels^32^, and Scipy libraries^33^.

### Bayesian network estimation and analysis

The basic analysis explained in the previous section showed the relationship between influenza onset and each background factor. To obtain deeper insights, we aimed to extract the causal relationships among the variables, including the causal pathways toward influenza onset. To this end, we performed a Bayesian network (BN) analysis.

A BN is a probabilistic graphical model that represents the conditional independence between multiple variables as a directed acyclic graph (i.e., a network). The directed edges (arcs) in the estimated network can be interpreted as causal relationships between the variables. BNs have been successfully used to model a wide variety of systems, including gene regulatory networks^34–39^, transcriptome networks^40^, protein signaling networks^41,42^, and medical diagnosis processes^43–46^.

Let $\:{X}_{1},\cdots\:,{X}_{p}$ be random variables (corresponding to each health checkup item). In a BN, each variable is represented by a node (vertex). The joint probabilistic density of $\:{X}_{1},\cdots\:,{X}_{p}$ can be given as

1 $$\:\begin{array}{c}f\left({X}_{1},\cdots\:,{X}_{p};{\varvec{\theta\:}}_{G}\right)=\prod\:_{j=1}^{p}f\left({X}_{j}|{X}_{{j}_{1}},\cdots\:,{X}_{{j}_{{q}_{j}}};{\varvec{\theta\:}}_{j}\right),\end{array}$$

where $\:{X}_{{j}_{1}},\cdots\:,{X}_{{j}_{{q}_{j}}}$ are variables dependent of $\:{X}_{j}$, $\:{\varvec{\theta\:}}_{j}$ is a parameter vector of the model for $\:{X}_{j}$, and $\:{\varvec{\theta\:}}_{G}={\left({\varvec{\theta\:}}_{1}^{\text{T}},\cdots\:,{\varvec{\theta\:}}_{p}^{\text{T}}\right)}^{\text{T}}$ is a parameter matrix of the network $\:G$ modeling the joint distribution of $\:{X}_{1},\cdots\:,{X}_{p}$. In a BN, this decomposition is represented by a directed acyclic graph. Each variable $\:{X}_{j}\:\left(j=1,\cdots\:,p\right)$ corresponds to a node, and the nodes $\:{X}_{{j}_{1}},\cdots\:,{X}_{{j}_{{q}_{j}}}$ are parents of the node $\:{X}_{j}$, which means that directed edges (arcs) are respectively drawn from the nodes $\:{X}_{{j}_{1}},\cdots\:,{X}_{{j}_{{q}_{j}}}$ to the node $\:{X}_{j}$.

We used the $\:B$-spline nonparametric regression version of the BN^34^, which allows flexible modeling of the nonlinear continuous relationship between variables. It models the variable $\:{x}_{j}\sim\:{X}_{j}\:(j=1,\cdots\:p)$ using $\:B$-spline curves with a noise term as follows.

2 $$\:\begin{array}{c}{x}_{j}={m}_{1}^{\left(j\right)}\left({x}_{{j}_{1}}\right)+\cdots\:+{m}_{{q}_{j}}^{\left(j\right)}\left({x}_{{j}_{{q}_{j}}}\right)+{\epsilon\:}_{j},\end{array}$$

where

3 $$\:\begin{array}{c}{m}_{k}^{\left(j\right)}\left(x\right)=\sum\:_{m=1}^{M}{\gamma\:}_{j,k,m}{b}_{j,k,m}\left(x\right)\:\left(k=1,\cdots\:,{q}_{j}\right)\end{array}$$

is a regression function using $\:M$ $\:B$-spline basis functions $\:{b}_{j,k,1},\cdots\:,{b}_{j,k,M}$ and their coefficients $\:{\gamma\:}_{j,k,1},\cdots\:,{\gamma\:}_{j,k,M}$, and $\:{\epsilon\:}_{j}\sim\:N\left(0,{\sigma\:}_{j}^{2}\right)$ is a normally-distributed noise term. In this model, the parameter vector $\:{\varvec{\theta\:}}_{j}$ is composed of $\:{\gamma\:}_{j,k,m}\:(k=1,\cdots\:,{q}_{j},\:\:m=1,\:\cdots\:,M)$ and $\:{\sigma\:}_{j}^{2}$.

To estimate the network structure, that is, the conditional independence between the variables described in Eq. (1), we adopted an approach that maximizes the posterior probability $\:p\left(G|X\right)$, where $\:G$ is the network structure and $\:X$ is the $\:n$-by-$\:p$ observed data matrix ($\:n$ and $\:p$ are the number of samples and variables, respectively). We denote the $\:j$-th variable with respect to the $\:i$-th sample (i.e., the $\:(i,j)$-element of $\:X$) as $\:{x}_{i,\:j}$. As indicated by Imoto et al.^34^, this optimization can be achieved by maximizing the following criteria:

4 $$\:\begin{array}{c}{\pi\:}_{G}\int\:\prod\:_{i=1}^{n}f\left({x}_{i,1},\cdots\:,{x}_{i,p};{\varvec{\theta\:}}_{G}\right)\pi\:\left({\varvec{\theta\:}}_{G}|\varvec{\lambda\:}\right)d{\varvec{\theta\:}}_{G},\end{array}$$

where $\:{\pi\:}_{G}$ is the prior probability of $\:G$, and $\:\pi\:\left({\varvec{\theta\:}}_{G}|\varvec{\lambda\:}\right)$ is the prior distribution of the unknown network parameter vector $\:{\varvec{\theta\:}}_{G}$ with the hyperparameter vector $\:\varvec{\lambda\:}$. To determine the optimal network structure that maximizes the criterion described in Eq. (4), we adopted an algorithm based on a greedy hill-climbing strategy and bootstrapping^35^. The constraints were applied such that the node representing influenza onset had no child nodes. In addition, we pruned edges with a bootstrap probability of less than 0.05. The above BN estimations were performed using the INGOR software^47,48^.

After determining the structure of the BN, we extracted network profiles representing participant-specific influenza-related characteristics. For this type of analysis, Tanaka et al.^47^ proposed a feature called the edge contribution value (ECv). The ECv of an edge from the $\:{j}_{k}$-th node (the $\:k$-th parent node of the $\:j$-th node) to the $\:j$-th node with respect to the $\:i$-th sample (participant) is defined as

5 $$\:\begin{array}{c}{\text{E}\text{C}\text{v}}_{i}\left({j}_{k}\to\:j\right)={m}_{k}^{\left(j\right)}\left({x}_{i,{j}_{k}}\right).\end{array}$$

As described in Eq. (2), the value of $\:j$-th variable is modeled as the sum of the regression functions $\:{m}_{k}^{\left(j\right)}\:\left(k=1,\cdots\:,{q}_{j}\right)$, where $\:{q}_{j}$ is the number of the parents. Therefore, $\:{\text{E}\text{C}\text{v}}_{i}\left({j}_{k}\to\:j\right)$ can be interpreted as the weight of the edge $\:{j}_{k}\to\:j$, i.e., how importantly the $\:k$-th parent contributes to the $\:j$-th node. An ECv and its difference, called $\:{\Delta\:}$ECv, facilitate the quantitative comparison of two (groups of) samples based on the network and the extraction of the edges that are differently regulated between the two samples (or groups). Previous studies^39,47,49^ have successfully extracted disease-specific characteristics in gene expression and clinical findings using ECvs and/or $\:{\Delta\:}$ECvs.

Despite the effectiveness of ECvs, their possible values may vary from one sample to another. Therefore, ECvs may not be suitable for extracting individual characteristics from a BN. To solve this problem, Tanaka et al.^38^ proposed a relative contribution (RC) value. An RC value is defined as the absolute value of the ECv normalized to the maximum ECv of the edges toward the same node. Formally, the RC value of the edge $\:{j}_{k}\to\:j$ with respect to the $\:i$-th sample is defined as

6 $$\:\begin{array}{c}{\text{R}\text{C}}_{i}\left({j}_{k}\to\:j\right)=\frac{\left|{\text{E}\text{C}\text{v}}_{i}\left({j}_{k}\to\:j\right)\right|}{\underset{1\le\:{k}^{{\prime\:}}\le\:{q}_{j}}{\text{max}}\left|{\text{E}\text{C}\text{v}}_{i}\left({j}_{{k}^{{\prime\:}}}\to\:j\right)\right|},\end{array}$$

where $\:{q}_{j}$ is the number of parents in the $\:j$-th node. Clearly, the possible RC values range from zero to one, regardless of the sample. Therefore, an RC value provides a better network profile for obtaining sample-specific insights.

Although RC values are useful network profiles, they are defined on individual parent-child edges and cannot be directly applied to a sequence of edges, that is, a causal pathway. To evaluate the importance of each pathway, we propose a novel criterion: the PathRC value. This is a multiplication of the RC values of all the edges contained in the pathway. Formally, the PathRC value of a length-$\:l$ pathway $\:{j}_{1}\to\:{j}_{2}\to\:\cdots\:\to\:{j}_{l+1}$ with respect to the $\:i$-th sample is defined as

7 $$\:\begin{array}{c}{\text{P}\text{a}\text{t}\text{h}\text{R}\text{C}}_{i}\left({j}_{1}\to\:{j}_{2}\to\:\cdots\:\to\:{j}_{l+1}\right)=\prod\:_{{l}^{{\prime\:}}=1}^{l}{\text{R}\text{C}}_{i}\left({j}_{{l}^{{\prime\:}}}\to\:{j}_{{l}^{{\prime\:}}+1}\right).\end{array}$$

PathRC values are useful profiles for estimating participant-wise differences in the importance of each pathway to influenza onset. In addition, because pathways with sufficiently low PathRC values for all samples can be considered unimportant, we can prune them for simple and easy-to-understand visualization. This procedure facilitates a better understanding of the network structure.

In this study, we calculated the PathRC values for all pathways to influenza onset with respect to all participants after determining the BN structure. We pruned pathways with PathRC values lower than the predefined threshold $\:-{\text{log}}_{2}0.3\:\left(\approx\:1.737\right)$ for all participants.

Using this simplified network, we computed the participant-wise network profile vectors composed of RC values for all edges. Cluster analysis was performed based on the network profiles. Ward’s method was used for agglomerative hierarchical clustering. To identify risk factors specific to each cluster, we compared each cluster against all other clusters combined (e.g., Cluster 1 vs. others, Cluster 2 vs. others, …). For each comparison, we applied the bootstrap method (1,000 iterations) to estimate 95% confidence intervals, and extracted features whose intervals did not include 0 as cluster-specific risk factors. We defined the identified cluster-specific factors that were also identified in our basic analysis as “main factors,” except for those with inconsistent susceptibility. In addition, we obtained cluster-specific subgraphs by extracting the paths from main factors to influenza onset. These analyses were performed using the Python and Seaborn library^50^.

## Supplementary Information

Below is the link to the electronic supplementary material.

Supplementary Material 1

## Data Availability

The datasets generated and/or analysed during the current study are not publicly available due to privacy concerns but are available from the corresponding author on reasonable request.
