# Supplementary material for: Network analysis reveals causal relationships among individual background risk factors leading to influenza susceptibility
Source: Sci Rep. 2025 Aug 21;15:30721. doi: 10.1038/s41598-025-15131-4 (PMC12370951; doi:10.1038/s41598-025-15131-4)
Supplement: Supplementary file 1 — Supplementary Material 1 [file 41598_2025_15131_MOESM1_ESM.docx]

*Supplementary information*

Network analysis reveals causal relationships among individual background risk factors leading to influenza susceptibility

Akihide Terada, Kenji Fujimoto, Kazuyoshi Kise, Kenta Fujiwara, Eiichiro Uchino,
Yutaka Mizuma, Yoshinori Nishioku, Kenzo Takahashi, Ken Itoh, Tatsuya Mikami,
Koichi Murashita, Shigeyuki Nakaji, Yukihiro Fujita, Yasushi Okuno, and Yoshinori Tamada


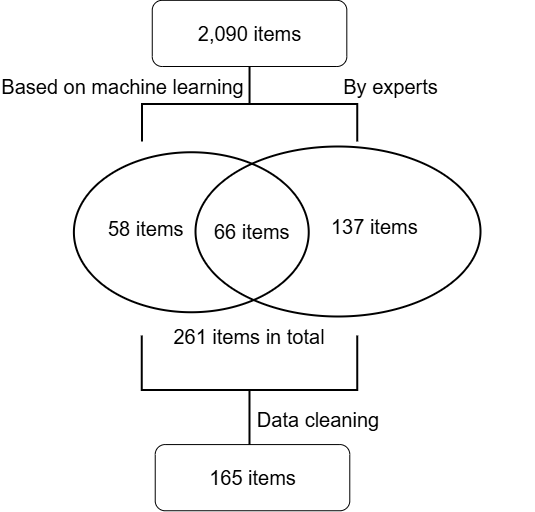


Supplementary Figure S1: Overview of the item selection procedure


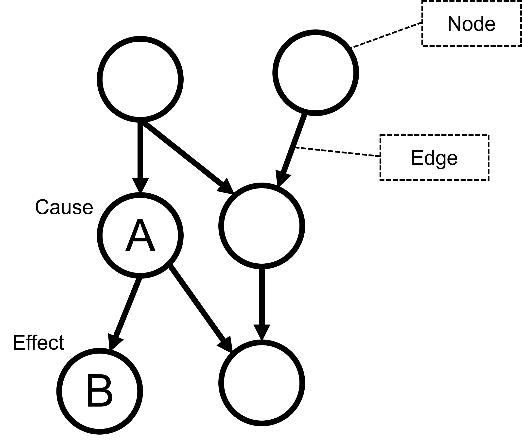


Supplementary Figure S2: An example of a network. Each node represents a variable, and each edge (directed arrow) represents dependency (cause-effect or influence relationship). Within this network, there is an edge from node A to node B. A is called a parent of B, and B is a child of A. As shown in figure, a directed arrow from node A to node B indicates that node A has a direct probabilistic influence on node B, and that the probability distribution of B is conditionally dependent on the state of A.


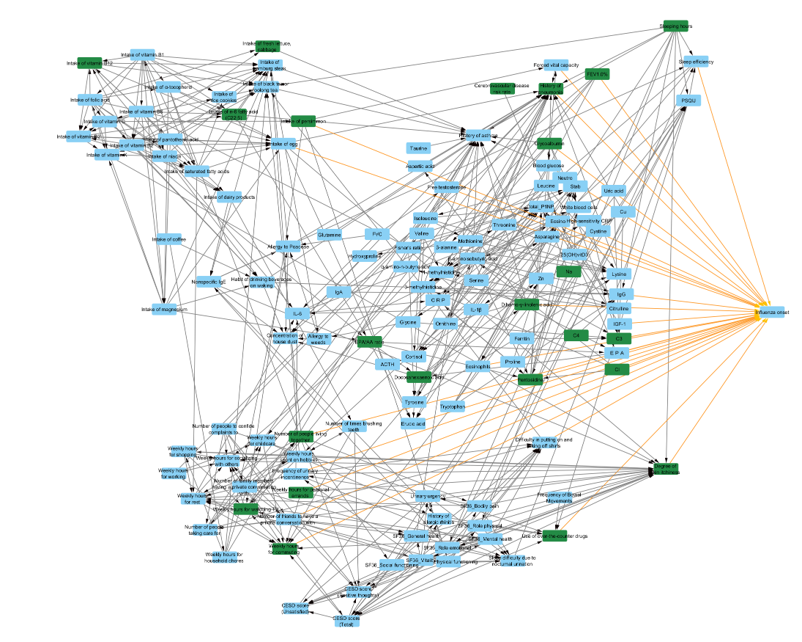


Supplementary Figure S3: A network leading to influenza onset obtained through network structure estimation. The items represented by green nodes were identified in the basic analysis. Orange edges are direct connections toward “Influenza onset.” An interactive version of the network is publicly available at this URL: https://www.ndexbio.org/viewer/networks/7dbbd321-51bf-11f0-a218-005056ae3c32

Supplementary Table S1: Causal pathways of the factors identified in our basic analysis of influenza onset extracted from our Bayesian network analysis

| Factor | Pathway to influenza |
| --- | --- |
| History of pneumonia | History of Pneumonia → Influenza onset |
| Glucose-related index | Glycoalbumin and pentosidine → White blood cells → Eosinophils → Influenza onset |
|  | Pentosidine → CRP quantification → High-sensitivity CRP → C3 → Influenza onset |
|  | Glycoalbumin → C3 → Influenza onset |
| Sodium | Na → Pentosidine and total P1NP → Influenza onset |
| Chlorine | Cl → Pentosidine → Influenza onset |
| Lipid | EPA/AA ratio → Pentosidine, EPA → Influenza onset |
|  | Dihomo-γ-linolenic acid → Bronchial asthma → History of pneumonia → Influenza onset |
| Sleep | Sleeping hours → History of pneumonia → Influenza onset |
|  | Sleeping hours → IgG → Influenza onset |
| Immunity | IL-6 → CRP quantification → High-sensitivity CRP → C3 → Influenza onset |
|  | Ferritin, glycine, and Cu → C3 → Influenza onset |
|  | C4 → IgG → Influenza onset |
| Allergy | Allergy → Allergic rhinitis → Use of over-the-counter medicines → Influenza onset |
| Nutrient intake | Intake of persimmon → Influenza onset |
|  | Intake of vitamin B12 → IL-6 → White blood cells → Eosinophils → Influenza onset |
| Living environment | Number of people living together → Influenza onset |

Supplementary Table S2: Items with significant differences between Clusters 1 and 2-5 identified in the RC value-based clustering of all participants (corresponding to Figure 2a)

| Domain | Item | Cluster1 (Mean ± SD) | Others (Mean ± SD) | Averaged difference [95% CI] | Susceptibility to influenza |
| --- | --- | --- | --- | --- | --- |
| Background | History of pneumonia^†^ | 0.62 ± 0.49 | 0.01 ± 0.10 | 0.61 [0.50 to 0.71] | Having history and/or complications |
|  | History of asthma | 0.15 ± 0.36 | 0.04 ± 0.20 | 0.11 [0.04 to 0.19] | Having history and/or complications |
|  | Alcohol history | 1.71 ± 0.56 | 1.55 ± 0.57 | 0.16 [0.04 to 0.29] | Having history |
| Environment | Number of people taking care for | 2.28 ± 3.39 | 1.55 ± 1.59 | 0.73 [0.02 to 1.63] | Many |
|  | Sleeping hours | 6.46 ± 1.40 | 6.94 ± 1.11 | -0.49 [-0.81 to -0.16] | Short |
|  | Sleeping hours | 92.84 ± 13.90 | 97.08 ± 5.96 | -4.30 [-7.63 to -1.26] | Inefficient |
|  | PSQJ^†^ | 4.68 ± 2.93 | 3.61 ± 2.14 | 1.08 [0.39 to 1.79] | High |
|  | Intake of dairy products  (yes:1 / no: 0) | 0.61 ± 0.49 | 0.46 ± 0.50 | 0.15 [0.05 to 0.26] | Frequent |
| Blood test | Glycoalbumin | 15.63 ± 4.10 | 14.51 ± 1.65 | 1.14 [0.35 to 2.15] | High |
|  | Behenic acid | 1.46 ± 0.40 | 1.58 ± 0.50 | -0.12 [-0.21 to -0.03] | Low |
|  | Erucic acid | 0.06 ± 0.08 | 0.04 ± 0.03 | 0.02 [0.00 to 0.04] | High |
|  | Urinary albumin | 16.94 ± 41.28 | 34.62 ± 203.10 | -17.95 [-33.61 to -2.98] | Low |
|  | Interleukin 6 | 1.24 ± 0.99 | 1.86 ± 6.11 | -0.62 [-1.10 to -0.25] | Low |
|  | CRP^†^ | 0.06 ± 0.07 | 0.13 ± 0.48 | -0.07 [-0.11 to -0.04] | Low |
|  | High-sensitivity CRP | 0.05 ± 0.06 | 0.07 ± 0.11 | -0.02 [-0.03 to -0.01] | Low |
|  | Weeds sensitization | 0.23 ± 0.43 | 0.40 ± 1.49 | -0.17 [-0.31 to -0.04] | Low |
|  | β－alanine | 344.99 ± 74.82 | 327.09 ± 79.32 | 18.39 [0.47 to 36.21] | High |
| Nutrient intake | Intake of rice cookies | 4.89 ± 1.37 | 4.48 ± 1.37 | 0.40 [0.07 to 0.74] | Few |

“Intake of rice cookies” is higher for the lower amount of intake.

Variables marked with † are statistically significant after Dunn-Šidák correction for multiple hypothesis comparison [14].

Supplementary Table S3: Items considered to increase the onset rate found by logistic regression analysis for Clusters 2-5 identified in the RC value-based clustering of all participants (corresponding to Figure 2a)

| Domain | Item | Odds ratio [95% CI] | Susceptibility to influenza |
| --- | --- | --- | --- |
| Environment | Number of people living together | 1.20 [1.07 to 1.36] | High |
|  | Number of friends to have a private conversation with | 0.85 [0.75 to 0.95] | Low |
|  | Weekly hours for commuting | 1.03 [1.01 to 1.05] | Long |
|  | Use of over-the-counter drugs | 1.80 [1.02 to 3.18] | Using |
|  | Intake of dairy products  (yes:1 / no: 0) | 0.62 [0.40 to 0.96] | No |
|  | Difficulty in taking on and off the clothes | 2.17 [1.11 to 4.24] | Difficult |
| Blood test | Na | 0.79 [0.70 to 0.89] | Low |
|  | Cl | 0.86 [0.78 to 0.96] | Low |
|  | C4 | 0.95 [0.91 to 0.98] | Low |
| Nutrient intake | Intake of persimmon | 1.26 [1.09 to 1.46] | Low |
| Other | FEV1.0% | 1.04 [1.01 to 1.08] | High |

“Intake of rice cookies” is higher for the lower amount of intake.

Variables marked with † are statistically significant after Dunn-Šidák correction for multiple hypothesis comparison [14].

Supplementary Table S4: Influenza onset rates in each cluster identified by hierarchical clustering of all cases without RC values (corresponding to Figure 2b)

| Cluster | Not infected | Infected | Total |
| --- | --- | --- | --- |
| 1 | 345 (87.1%) | 51 (12.9%) | 396 |
| 2 | 268(89.0%) | 33 (11.0%) | 301 |
| 3 | 187 (89.5%) | 22 (10.5%) | 209 |
| 4 | 141 (90.4%) | 15 (9.6%) | 156 |
| total | 941 | 121 | 1062 |

Supplementary Table S5: Significant variables identified in the RC value-based clustering of participants with influenza. Items showing statistically significant differences in comparisons between each cluster and all other clusters combined (e.g., Cluster 1 vs. others, Cluster 2 vs. others, …) are listed.

| Cluster | items |
| --- | --- |
| Cluster 1 (n = 11) | Sex (Male:1, Female:0)$\uparrow$ |
|  | Number of family members having a private conversation with$\uparrow$ |
|  | Number of people to confide complaints to$\uparrow$ |
|  | Weekly hours spent on hobbies$\uparrow$ |
|  | *CESD score (Unsatisfied)$\uparrow$ |
|  | Blood glucose$\uparrow$ |
|  | **Glycoalbumin**$\uparrow$ |
|  | Erucic acid$\uparrow$ |
|  | Free testosterone$\uparrow$ |
|  | Ferritin$\uparrow$ |
|  | **Pentosidine**$\uparrow$ |
|  | Aspartic acid$\uparrow$ |
|  | Cortisol$\uparrow$ |
|  | **Intake of persimmon**$\uparrow$ |
|  | **Cerebrovascular disease risk rate**$\uparrow$ |
|  | β-alanine$\uparrow$ |
|  | Valine$\uparrow$ |
|  | Isoleucine$\uparrow$ |
|  | Leucine$\uparrow$ |
|  | Ethanolamine$\uparrow$ |
|  | 3-methylhistidine$\uparrow$ |
|  | Forced vital capacity$\uparrow$ |
| Cluster 2 (n = 15) | **Weekly hours for personal errands**$\uparrow$ |
|  | **History of pneumonia**$\uparrow$ |
|  | Total P1NP$\uparrow$ |
|  | Urine osmolarity$\uparrow$ |
|  | Intake of green tea$\uparrow$ |
| Cluster 3 (n = 9) | **Weekly hours for personal errands**$\uparrow$ |
|  | **Weekly hours for commuting**$\uparrow$ |
|  | **Sleeping hours**$\boldsymbol{\downarrow}$ |
|  | Sleep efficiency$\boldsymbol{\downarrow}$ |
|  | PSQIJ [21]$\uparrow$ |
|  | Sleep difficulty due to nocturnal urination$\uparrow$ |
|  | Frequency of urinary incontinence$\uparrow$ |
|  | Glycine$\boldsymbol{\downarrow}$ |
|  | **SF36_Physical functioning$\boldsymbol{\downarrow}$ |
|  | **SF36_Role physical$\boldsymbol{\downarrow}$ |
|  | **SF36_Vitality$\boldsymbol{\downarrow}$ |
|  | Intake of vitamin B1$\uparrow$ |
|  | Intake of vitamin C$\uparrow$ |
|  | Intake of vitamin fatty acids$\uparrow$ |
| Cluster 4 (n = 5) | Weekly hours for household chores$\uparrow$ |
|  | Degree of skin itchiness$\boldsymbol{\downarrow}$ |
|  | Intake of rice cookies$\uparrow$ |
|  | **Intake of fresh lettuce, cabbage**$\boldsymbol{\downarrow}$ |
|  | Intake of egg$\boldsymbol{\downarrow}$ |
|  | Proline$\uparrow$ |
|  | **SF36_Mental health$\uparrow$ |
|  | Intake of black tea or oolong tea$\uparrow$ |
| Cluster 5 (n = 16) | Weekly hours for watching TV$\uparrow$ |
|  | *CESD score (Positive thoughts) $\uparrow$ |
|  | **Degree of skin itchiness**$\uparrow$ |
|  | White blood cells$\uparrow$ |
|  | Eosinophils$\uparrow$ |
|  | Forced vital capacity$\uparrow$ |
| Cluster 6 (n = 54) | Weekly hours for personal errands$\boldsymbol{\downarrow}$ |
|  | **Weekly hours for watching TV**$\boldsymbol{\downarrow}$ |
|  | Weekly hours spent on hobbies$\boldsymbol{\downarrow}$ |
|  | Weekly hours for working$\uparrow$ |
|  | History of pneumonia$\boldsymbol{\downarrow}$ |
|  | Use of over-the-counter drugs$\boldsymbol{\downarrow}$ |
|  | Degree of skin itchiness$\boldsymbol{\downarrow}$ |
|  | Urine osmolarity$\boldsymbol{\downarrow}$ |
|  | White blood cells$\boldsymbol{\downarrow}$ |
|  | Cortisol$\boldsymbol{\downarrow}$ |
|  | Blood glucose$\boldsymbol{\downarrow}$ |
|  | FEV1.0%$\uparrow$ |
|  | Cerebrovascular disease risk rate$\boldsymbol{\downarrow}$ |
|  | Nonspecific IgE$\boldsymbol{\downarrow}$ |
|  | Eosinophils$\boldsymbol{\downarrow}$ |
|  | Ethanolamine |
|  | Proline$\boldsymbol{\downarrow}$ |
|  | β-alanine$\boldsymbol{\downarrow}$ |
|  | Isoleucine$\boldsymbol{\downarrow}$ |
|  | Phenylalanine$\boldsymbol{\downarrow}$ |
|  | Intake of vitamin C$\boldsymbol{\downarrow}$ |
|  | Forced vital capacity$\boldsymbol{\downarrow}$ |
| Cluster 7 (n = 10) | **Use of over-the-counter drugs**$\uparrow$ |
|  | IL-6$\uparrow$ |
|  | IL-1β$\uparrow$ |
|  | CRP$\uparrow$ |
|  | Nonspecific IgE$\uparrow$ |
|  | Allergy to Poaceae$\uparrow$ |
|  | Allergy to weeds$\uparrow$ |
|  | Concentration of house dust$\uparrow$ |
|  | Concentration of ceda$\uparrow$ |
|  | **Ankle-brachial pressure index**$\uparrow$ |

$\uparrow$ and $\downarrow$ indicate the susceptibility, i.e., higher and lower values of the corresponding predictor are associated with increased risk of influenza, respectively. Items identified in our basic analysis are shown in **bold**. Items with susceptibility inconsistent with our basic analysis are not shown in bold.

* CESD is a short self-report scale developed from items used in previously developed depression scales.

Jiang, L. *et al*. The Reliability and Validity of the Center for Epidemiologic Studies Depression Scale (CES-D) for Chinese University Students. *Front Psychiatry*. **10,** 315 (2019).

** SF-36 is a 36-item questionnaire that measures health-related quality of life across eight domains, including physical and mental health.

Ware, J. E., Sherbourne, C. D. The MOS 36-item short-form health survey (SF-36). I. Conceptual framework and item selection. *Med Care*. **30** (6), 473-83 (1992).
